# Macrophage mannose receptor CD206-targeted PET imaging in experimental acute myocardial infarction

**DOI:** 10.1186/s13550-025-01254-2

**Published:** 2025-06-04

**Authors:** Putri Andriana, Senthil Palani, Heidi Liljenbäck, Imran Iqbal, Vesa Oikonen, Jenni Virta, Konstantina Makrypidi, Johan Rajander, Erika Atencio Herre, Aino Suni, Sirpa Jalkanen, Juhani Knuuti, Luisa Martinez-Pomares, Ioannis Pirmettis, Xiang-Guo Li, Antti Saraste, Anne Roivainen

**Affiliations:** 1 Turku PET Centre, University of Turku, Turku, Finland; 2 Turku Center of Disease Modeling, University of Turku, Turku, Finland; 3 Institute of Nuclear and Radiological Science and Technology, Energy and Safety, NCSR “Demokritos”, Athens, Greece; 4 Turku PET Centre, Accelerator Laboratory, Åbo Akademi University, Turku, Finland; 5 MediCity Research Laboratory, University of Turku, Turku, Finland; 6 InFLAMES Research Flagship, University of Turku, Turku, Finland; 7 Turku PET Centre, Turku University Hospital, Kiinamyllynkatu 4-8, 20520 Turku, Finland; 8 School of Life Sciences, University of Nottingham, Nottingham, UK; 9 Department of Chemistry, University of Turku, Turku, Finland; 10 Heart Center, Turku University Hospital and University of Turku, Turku, Finland

**Keywords:** CD206, Inflammation, Macrophage mannose receptor, Myocardial infarction, PET

## Abstract

**Background:**

The macrophage mannose receptor (CD206) is expressed predominantly on the surface of M2-type macrophages, which play a role in resolution of inflammation after myocardial injury. The purpose of this study was to evaluate the utility of CD206-targeted PET tracer Al[^18^F]F-NOTA-D10CM, a fluorinated mannosylated dextran derivative, for imaging immune responses after experimental acute myocardial infarction (MI).

**Results:**

Flow cytometry revealed selective binding of Alexa-488-NOTA-D10CM to human M2-polarized macrophages derived from blood monocytes compared to M1 macrophages. The binding affinity of Al[^18^F]F-NOTA-DCM for CD206-positive Chinese hamster ovary cells was 1.83 ± 0.68 nM. In vivo PET and ex vivo autoradiography experiments in Sprague–Dawley rats studied at 3 and 7 days after permanent ligation of the left coronary artery or a sham-operation, showed significantly higher uptake of Al[^18^F]F-NOTA-DCM in the MI area than in remote areas, or the myocardium of sham-operated rats. However, there was no difference in uptake in the MI area between day 3 and day 7. Uptake of Al[^18^F]F-NOTA-DCM in the MI area correlated positively with the area-% of CD206-positive staining of the left ventricular myocardium (*r* = 0.481, *P* = 0.006). In vitro competition studies on tissue cryosections using a molar excess of unlabeled D10CM revealed a reduction of approximately 85%, confirming specific tracer binding.

**Conclusion:**

Al[^18^F]F-NOTA-D10CM PET detects overexpression of CD206 after ischemic myocardial injury, and may be a suitable biomarker for detecting M2-type macrophages associated with the inflammatory process post-MI.

**Graphical abstract:**

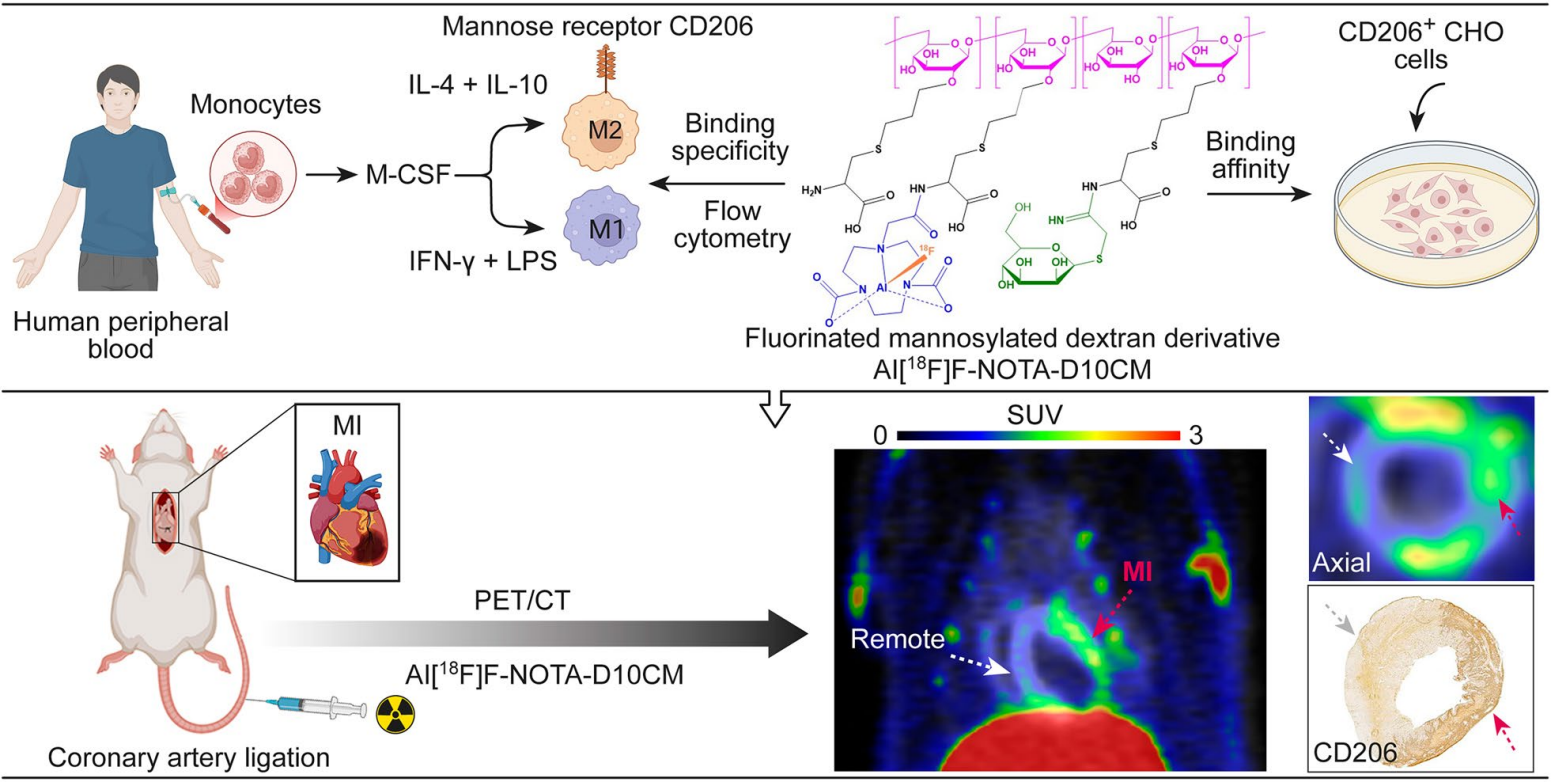

**Supplementary Information:**

The online version contains supplementary material available at 10.1186/s13550-025-01254-2.

## Introduction

Macrophages play a major role in determining the balance between pro-inflammatory and anti-inflammatory immune responses after myocardial infarction (MI) [1, 2]. Targeted imaging of different leukocyte subpopulations using positron emission tomography (PET) tracers may provide information about the dynamics of these immune responses [2]. Macrophage mannose receptor cluster of differentiation 206 (CD206), which is expressed predominantly by M2-type (alternatively activated, anti-inflammatory) macrophages, has attracted attention as a biomarker for PET. Radiotracers targeting CD206 have been approved for clinical use for sentinel lymph node mapping by noninvasive imaging and are being investigated for other applications [3–5]. Previously, mannose receptor targeted imaging has been evaluated in experimental models of atherosclerosis, MI and myocarditis [6–10]. Alternatively activated M2-type macrophages promote cardiac repair post-MI by regulating activation of fibroblasts [11].

We have described a new CD206-targeted PET tracer, an aluminum fluoride-18-labeled 1,4,7-triazacyclononane-1,4,7-triacetic acid (NOTA)-conjugated mannosylated dextran derivative (22 kDa) called Al[^18^F]F-NOTA-D10CM [12], and validated its target specificity and ability to detect inflammation in wild-type versus CD206 knockout mice [13]. In the present study, we evaluated the ability of Al[^18^F]F-NOTA-D10CM to detect CD206 in rats at the early stage post-MI.

## Materials and methods

### Preparation of NOTA-D10CM, Alexa-488-NOTA-D10CM, and Al[^18^F]F-NOTA-D10CM

NOTA-D10CM was synthesized in accordance with a published protocol [14]. In brief, D10CM in dimethyl sulfoxide was conjugated to NOTA-*N*-hydroxysuccinimide ester in borate buffer for 18 h at room temperature, with stirring. The product was then diluted with deionized water, concentrated using an ultrafiltration cell under nitrogen gas pressure, and lyophilized to yield a white solid product.

The NOTA-D10CM was labeled with the near infrared dye Alexa Fluor® 488 tetrafluorophenyl ester (Alexa Fluor® 488 Microscale Protein Labeling Kit A3006; Invitrogen) according to the manufacturer’s protocol. Briefly, to make 1 mg/mL Alexa-488-NOTA-D10CM, a reaction tube containing 100 µL of a 1 mM stock solution of NOTA-D10CM was supplemented with 10 µL of 1 M sodium bicarbonate. The mixture was then homogenized manually using a micropipette. To make a stock solution of reactive dye, 10 µL of deionized water were added to a vial containing the Alexa Fluor® 488 tetrafluorophenyl ester provided in the kit, resulting in a reactive dye solution with a concentration of 11.3 nmol/µL. Subsequently, 8 µL of reactive dye solution was added to the reaction tube containing NOTA-D10CM in sodium bicarbonate and then incubated for 15 min at room temperature. The unreacted dye was removed from the mixture using the purification resin and spin filters provided in the labeling kit.

Al[^18^F]F-NOTA-D10CM was prepared according to a previously published method [12]. Briefly, the Al[^18^F]F-fluorination technique was used to radiolabel NOTA-D10CM (6.8 nmol in 50 µL water) with [^18^F]fluoride (220 µL in saline) by heating at 100⁰C for 13 min in a mixture of AlCl_3_/1 M sodium acetate buffer (pH 4.0, 40 µL), acetonitrile (60 µL), and 150 mM ascorbic acid (40 µL); the mixture was then cooled to 40⁰C. Trifluoroacetic acid (TFA) in water (1%, 810 µL) was then added to the reaction mixture, the whole mixture was purified following the previously described method [12]. Al[^18^F]F-NOTA-D10CM was collected in an end product bottle containing a mixture of 0.15 mL of 150 mM ascorbic acid and 1.35 mL phosphate-buffered saline (PBS). The radiochemical purity was measured using a radiodetector-coupled high-performance liquid chromatography (HPLC) tandem system (Hitachi; Merck), as previously described [12].

### Binding of Alexa-488-NOTA-D10CM to M1/M2-polarized human macrophages

Peripheral blood mononuclear cells (PBMCs) were isolated from human buffy coats by Ficoll centrifugation. Monocytes were enriched from PBMCs by positive selection using a magnetic-activated cell sorting kit (Monocyte isolation kit with CD14 MicroBeads; Miltenyi Biotec). The monocytes were then cultured and polarized into M1 or M2 macrophages as previously described [15]. To assess CD206 expression, cells were pre-blocked with human immunoglobulin (Ig 100 μg/mL; KIOVIG, Baxter) and then incubated with an Alexa Fluor® 647-conjugated anti-human CD206 antibody (mouse IgG1; BioLegend) or an isotype control (mouse IgG1; BD Biosciences). Post-incubation, cells were fixed with paraformaldehyde and analyzed by flow cytometry (Fortessa, BD Biosciences) and Flowing software (Turku Center of Biotechnology). To evaluate binding of Alexa-488-NOTA-D10CM, macrophages were harvested and incubated for 30 min or 4 h at 37 °C in a CO_2_ incubator with fresh medium (Iscove’s Modified Dulbecco’s medium containing 2% AB serum and 2 mmol L-glutamine; Gibco, Thermo Fisher Scientific) containing Alexa-488-NOTA-D10CM (10 µg/mL). After incubation, the cells were rinsed twice with 1 mL of PBS to remove any unbound Alexa-488-NOTA-D10CM. Finally, cells were fixed using paraformaldehyde and analyzed by flow cytometry and Flowing software.

### Binding of Al[^18^F]F-NOTA-D10CM to CHO cells

Chinese hamster ovary (CHO) cells stably expressing mouse CD206 (CHO-CD206^+^) and CD206-negative CHO cells (CHO-CD206^−^) were a kind gift from Prof. Luisa Martinez-Pomares (University of Nottingham, United Kingdom). The cells were cultured at 37 °C in a CO_2_ incubator in RPMI 1640 medium (Gibco/Thermo Fisher Scientific) supplemented with 10% fetal bovine serum (Biowest), 2 mmol l-glutamine; (Gibco/Thermo Fisher Scientific), 100 U/mL penicillin, and 100 µg/mL streptomycin (Sigma-Aldrich/Merck). To validate CD206 expression, the cells were harvested and then incubated with an Alexa Fluor® 488-conjugated anti-mouse CD206 antibody (rat IgG2; Bio-Rad) or with an isotype control (rat IgG2; BD Biosciences). Then, the cells were fixed using paraformaldehyde and analyzed using flow cytometer and Flowing software.

To study the binding affinity of Al[^18^F]F-NOTA-D10CM, CHO-CD206^+^ and CHO-CD206^−^ cells were cultured and allowed to attach to opposite sides of a 92 mm Petri dish in accordance with the LigandTracer (Ridgeview Instruments AB) guidelines. An empty region on the Petri dish (with no attached cells) was used as a background control for non-specific binding of Al[^18^F]F-NOTA-D10CM. The dissociation constant (K_*D*_) of Al[^18^F]F-NOTA-D10CM was measured using a LigandTracer Yellow instrument, where the assay protocol includes sequential measurement of radioactivity in the target cells (CHO-CD206^+^), the negative control cells (CHO-CD206^−^), and the cell-free background regions of the Petri dish. Radioactivity in each region was measured for 30 s (as raw counts per second (cps)) with a 5 s delay over the time course of the experiment. The target regions (cps) were corrected for background signals and radioactive decay. Al[^18^F]F-NOTA-D10CM was added to the medium in a stepwise fashion to achieve a concentration range of 50–450 nM, followed by replacement with fresh medium without tracer to measure the dissociation. The ratio of bound Al[^18^F]F-NOTA-D10CM (to the cells) to background (Petri dish) and the K_*D*_ value were calculated using the TraceDrawer software (Ridgeview Instruments AB).

### Animals and experimental design

All animal experiments were approved by the National Project Authorization Board in Finland (license number ESAVI/43134/2019) and carried out in compliance with the EU Directive 2010/EU/63 on the protection of animals used for scientific purposes.

MI was induced in male Sprague–Dawley rats by permanent ligation of the left anterior descending (LAD) coronary artery [16]. In brief, approximately 30 min prior to anesthesia, rats were subcutaneously (s.c.) medicated with 0.5 mg/kg buprenorphine (Temgesic 0.1 mg/kg, Schering-Plough); anesthesia was induced by intraperitoneal (i.p.) administration of a mixture of 80 mg/kg ketamine (Ketaminol®vet 50 mg/mL, Intervet) and 0.5 mg/kg medetomidine (Cepetor vet 50 mg/mL, Vetmedic). The thoracic area was shaved, cleaned, and treated with s.c. < 7 mg/kg lidocaine (Lidocaine, 10 mg/mL; Orion Pharma). The body temperature of the rats was maintained using a heating pad, and monitored with a digital thermometer. The rats were intubated and attached to a rodent ventilator (TOPO dual mode ventilator; Kent Scientific). All surgical equipment was sterilized, and the surgical area was kept sterile. LAD coronary artery was permanently ligated, and infarction was verified by the paleness of the myocardium. Sham-operated rats underwent the same procedure, except that the artery was not ligated. The incision was closed and the rats were revived by i.p. injection of 1 mg/kg atipamezole (Antisedan 5 mg/mL, Orion Pharma). Saline was administered s.c. before and after the surgery. The rats were monitored closely, and treated with buprenorphine every 8 h for 3 days following the operation. The mortality rate after the LAD ligation procedure was ~ 27% on MI Day 7, and ~ 55% on MI Day 3, mainly due to development of significant infarction on Day 0 after surgery or on Day 1 after surgery.

**Fig. 1:**
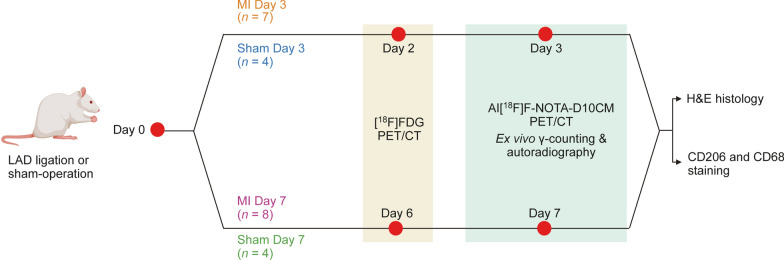
Animal study design. Sprague Dawley rats (*n* = 23) underwent permanent LAD ligation or sham-operation on Day 0. Then, the rats were divided to four groups: MI Day 3 (Group 1), Sham Day 3 (Group 2), MI Day 7 (Group 3), and Sham Day 7 (Group 4). All rats were imaged with [^18^F]FDG PET/CT 1 day before Al[^18^F]F-NOTA-D10CM PET/CT. All rats were euthanized immediately after Al[^18^F]F-NOTA-D10CM PET/CT imaging to measure ex vivo biodistribution, autoradiography, hematoxylin–eosin (H&E) staining, CD206 immunohistochemical staining, and double CD206 and CD68 immunofluorescence staining of the left ventricle

The experimental study design is depicted in Fig. 1. A total of 23 rats were divided into four groups. Group 1: MI Day 3 = 3 days post-MI (*n* = 7, weight 263.30 ± 56.00 g, age 7–8 weeks); Group 2: Sham Day 3 = 3 days after sham-operation (*n* = 4, 218.60 ± 18.00 g, 7 weeks); Group 3: MI Day 7 = 7 days post-MI (*n* = 10, 265.60 ± 28.50 g, 7–8 weeks); and Group 4: Sham Day 7 = 7 days after sham-operation (*n* = 4, 275.80 ± 6.00 g, 8 weeks). Rats underwent PET/computed tomography (CT) scans on consecutive days after intravenous (i.v.) injection of 2-deoxy-2-[^18^F]-fluoro-D-glucose ([^18^F]FDG; 34.90 ± 1.31 MBq) or Al[^18^F]F-NOTA-D10CM (50.51 ± 2.32 MBq [range 43.98–54.65 MBq], 0.29 ± 0.14 mg [range 0.12–0.63 mg], 13.39 ± 6.56 nmol [range 5.67–28.64 nmol]), followed by ex vivo analyses performed 70 min after Al[^18^F]F-NOTA-D10CM injection. Left ventricles were dissected, frozen, and cross-sectioned for digital autoradiography, histology (hematoxylin–eosin [H&E]), and anti-CD206 and anti-cluster of differentiation 68 (CD68) immunostaining.

### In vivo PET/CT studies

Rats were imaged with Inveon Multimodality PET/CT (Siemens Medical Solution) under isoflurane anesthesia (4–5% induction, 1.5–2% maintenance). The tail vein was cannulated before imaging. CT was performed for attenuation correction and anatomical reference. A 10-min static PET acquisition was performed 20 min after [^18^F]FDG injection to visualize the myocardium. A 60 min dynamic PET acquisition was started at the time of Al[^18^F]F-NOTA-D10CM injection. PET data were reconstructed using an ordered subsets expectation maximization 3-dimensional algorithm into 30 × 3 s, 9 × 10 s, 4 × 30 s, 5 × 60 s, and 10 × 300 s time frames. PET/CT images were analyzed using Carimas 2.10 software (Turku PET Centre, www.turkupetcentre.fi/carimas/). Regions of interest (ROI) within the main organs were defined manually using CT as a reference, and [^18^F]FDG PET to localize the myocardium. ROIs in the MI area were defined based on reduced [^18^F]FDG uptake on the short-axis images, and in the remote area based on strong [^18^F]FDG uptake in the septum, excluding the apical area to avoid spill-over from the liver. MI area ROIs were defined in each frame that showed visible Al[^18^F]F-NOTA-D10CM uptake. In addition, H&E stains were used to confirm the location of the MI and remote areas. At least three consecutive planes were analyzed at 50–60 min post-injection of Al[^18^F]F-NOTA-D10CM. Time-activity curves (TACs) were extracted from dynamic PET data and expressed as the mean standardized uptake value (SUV) as a function of time post-injection. Size of the MI was measured in [^18^F]FDG polar maps as myocardium showing < 70% tracer uptake of the maximum uptake.

### Modeling of PET data

The dynamic Al[^18^F]F-NOTA-D10CM PET data were analyzed using a graphical Patlak method, along with one- and two-tissue compartment models. The blood TACs obtained from the heart left ventricle cavity were used as an input function without metabolite-correction.

TACs were extracted from dynamic Al[^18^F]F-NOTA-D10CM PET data obtained from three manually defined ROIs: the MI area, an area remote from the infarction, and the left ventricle cavity. Due to the small target size, along with movement of the heart (beating, respiration), the TACs do not represent the pure intended target regions; rather, they are mixtures of the target regions and adjacent regions. This effect is particularly conspicuous in the myocardial regions during the first few minutes after injection, when the concentration of radioactivity in the heart cavities is very high. The effect is less prominent during the late phase of dynamic imaging (50–60 min post-injection), which is used to calculate the regional SUVs. During dynamic data analysis, the left ventricle cavity TAC was assumed to represent the concentration of intact Al[^18^F]F-NOTA-D10CM in arterial blood, ignoring the relatively low contribution of radioactive metabolites to the total concentration in the blood. The Patlak plot became linear about 8 min post-injection (results not shown), suggesting rapid kinetics, and that uptake by the myocardial muscle is irreversible, as to be expected for a radioligand that is internalized. Since the Patlak plot does not properly account for the highly variable volume of the heart cavity inside myocardial ROIs, compartmental models were applied to analyze the data. In these compartmental models, the regional TACs are assumed to be a composite of myocardial tissue (C_T) and blood (C_B),

$${\text{C}}\_{\text{ROI}}\left( {\text{t}} \right) = {\text{V}}\_{\text{B}}*{\text{C}}\_{\text{B}}\left( {\text{t}} \right) + \left( {{1} - {\text{V}}\_{\text{B}}} \right)*{\text{C}}\_{\text{T}}\left( {\text{t}} \right)$$

The one- and two-tissue compartment models tested showed a good fit to the data, but only the irreversible one-tissue compartment model with three parameters, K_i_, K_1_, and V_b_, provided consistent parameter estimates and so were used for further analyses.

### Ex vivo Al[^18^F]F-NOTA-D10CM studies

Rats were euthanized by cardiac puncture and cervical dislocation under isoflurane anesthesia 70 min after i.v. injection of Al[^18^F]F-NOTA-D10CM. Tissues of interest were excised, weighed, and their radioactivity measured using a gamma counter (Triathler 3 ˝; Hidex). The results were decay-corrected to the time of injection, compensated for radioactivity remaining in the tail, and expressed as a percentage of the injected radioactivity dose per gram of tissue (%ID/g).

The left ventricle was collected and washed with saline, embedded in Tissue-Tek OCT compound, frozen in dry ice-cooled isopentane, and cross-sectioned (serial 20 µm and 8 µm sections at approximately 1 mm intervals from the apex to base) using a cryostat (Leica CM3050 S, Leica Biosystems, Richmond Inc.); sections were then mounted on microscope slides (Leica Surgipath X-tra Adhesive, Leica Biosystems, Richmond Inc.) as described previously [17]. The slides were briefly air-dried, opposed to phosphor imaging plates (BAS-TR2025; Fuji), an exposed overnight. The plates were then scanned with a Fuji Analyzer BAS-5000. ROIs were analyzed on superimposed autoradiography and digitalized H&E histology images (20 µm sections) using Carimas. The results were expressed as photostimulated luminescence per square millimeter (PSL/mm^2^) decay-corrected to injection time and exposure time, and normalized to the injected radioactivity dose. All slides were stored in -70⁰C prior to H&E and immunohistochemical staining.

### In vitro binding of Al[^18^F]F-NOTA-D10CM to tissue sections

Cryosections (20 µm thickness) of the left ventricle were defrosted at 4 °C for 40 min, and placed in an incubation chamber for 15 min at room temperature in *N*-(2-hydroxyethyl)-piperazine-*N’*-(2-ethanesulfonic acid) (HEPES, Sigma-Aldrich) buffer pH 7.4 containing 10 mM Ca^2+^. For the total binding assay, slides were transferred to another chamber containing Al[^18^F]F-NOTA-D10CM (35 kBq/mL) in the HEPES buffer. For the competitive binding assay, adjacent tissue sections were incubated for 70 min with Al[^18^F]F-NOTA-D10CM (35 kBq/mL) and an approximately 200-fold molar excess of unlabeled NOTA-D10CM in the HEPES buffer. Then, the slides were rinsed twice with the cold HEPES buffer and dipped into cold water. The slides were briefly air-dried, exposed overnight to a phosphor imaging plate, scanned, and analyzed as described above. Experiments were performed in triplicate using tissue samples obtained from rats in MI Day 7 group (*n* = 3).

### Histology and immunostaining

Following autoradiography, frozen sections of left ventricle were stained with H&E (20 µm) as a histological reference, or with anti-CD206 (8 µm). Briefly, sections were fixed with 10% formalin, stained with hematoxylin (Fluka) and eosin (Reagena) using a Leica Autostainer, mounted in Pertex, and scanned with a digital slide scanner (Pannoramic P1000; 3DHistech Ltd.). For anti-CD206 immunohistochemical staining, sections were fixed in 4% paraformaldehyde, followed by antigen retrieval, washing, and blocking of endogenous peroxidase activity. Then, the sections were incubated for 60 min at room temperature with the polyclonal rabbit anti-mannose receptor (CD206/MRC1) antibody (working dilution 1:10,000; ab64693, Abcam), rinsed, and incubated with a secondary antibody (BrightVision horseradish peroxidase conjugated goat anti-mouse secondary antibody, DPVR110HRP, WellMed) for 30 min at room temperature. The sections were then reacted with 3,3-diaminobenzidine (BrightDAB, BS04-110, WellMed), counterstained with Mayer’s hematoxylin, mounted in Pertex, and dried overnight. Stained sections were scanned with a digital slide scanner (Pannoramic P1000 or Pannoramic 250 Flash, 3DHISTECH Ltd.), and examined using Pannoramic Viewer 1.15 software (3DHISTECH Ltd.) [12]. Quantitative analysis of the percentage CD206-positive area (CD206-positive area-%) were performed as described previously [13]. In brief, for quantitative analysis of the CD206-positive area-%, sections were separated to the MI area and the remote area using the manual selection tool in GIMP (version 2.10.24), using H&E histological reference to define the MI area (Supplementary Fig. 1). Quantification of the CD206-positive area-% in the MI and remote areas was performed separately by color deconvolution analysis using ImageJ 1.52n (Wayne Rasband), based on hematoxylin and DAB staining.

For double immunofluorescence staining, frozen left ventricle sections were incubated in citrate buffer (pH 6.0, BioSite) that was pre-warmed in boiling water. Then, the slides were cooled down for 20 min, washed with detergent (0.05% Tween 20)/PBS (pH 7.4), and pre-protein blocked (normal antibody diluent, BD09-125, WellMed). The slides were then incubated for 60 min at room temperature with mouse anti-rat CD68 (1:1000, MCA341GA, Bio-Rad) and polyclonal rabbit MRC-1 (1:10,000, ab64693, Abcam) primary antibodies in normal antibody diluent (BD09-125, WellMed). Subsequently, the sections were incubated with Alexa Fluor® 594-conjugated donkey anti-rabbit secondary antibody (A-21207, Invitrogen), and with an Alexa Fluor® 488-conjugated goat anti-mouse secondary antibody (A-11017, Invitrogen), for 30 min each at room temperature. Finally, the sections were mounted in Prolong Gold Antifade reagent with 4′,6-diamidino-2-phenylindole (DAPI, P36935, Invitrogen) and imaged with a Pannoramic Midi fluorescence slide scanner (3DHistech Ltd.). Data were analyzed using CaseViewer (version 2.4, 3DHistech Ltd.).

### Statistical analysis

Results are expressed as the mean ± standard deviation (SD). All data sets were first checked for normality using the D’Agostino-Pearson or Shapiro Wilk’s test. Differences between groups were determined by an unpaired Student’s *t* test, one-way Analysis of Variance (ANOVA), or the Wilcoxon test for non-normally distributed data. *P*-values < 0.05 were considered statistically significant. The association between two variables was evaluated by calculating Pearson’s correlation coefficient. All statistical analyses were conducted using GraphPad Prism (version 10.1.2 (324), 2023).

## Results

### Production of Al[^18^F]F-NOTA-D10CM

Al[^18^F]F-NOTA-D10CM was produced with the decay-corrected radiochemical yield of 18.57% ± 6.28. The amount of the obtained end product was 570.92 ± 211.91 MBq, starting from 5.62 ± 0.65 GBq of [^18^F]fluoride, resulting in a radioactivity concentration of 234.45 ± 73.76 MBq/mL and a molar activity of 9.87 ± 4.59 GBq/µmol at the end of synthesis (*n* = 13). The synthesis of Al[^18^F]F-NOTA-D10CM took 92.5 ± 10.9 min from the end of bombardment to the end of synthesis.

### Cell binding studies

**Fig. 2:**
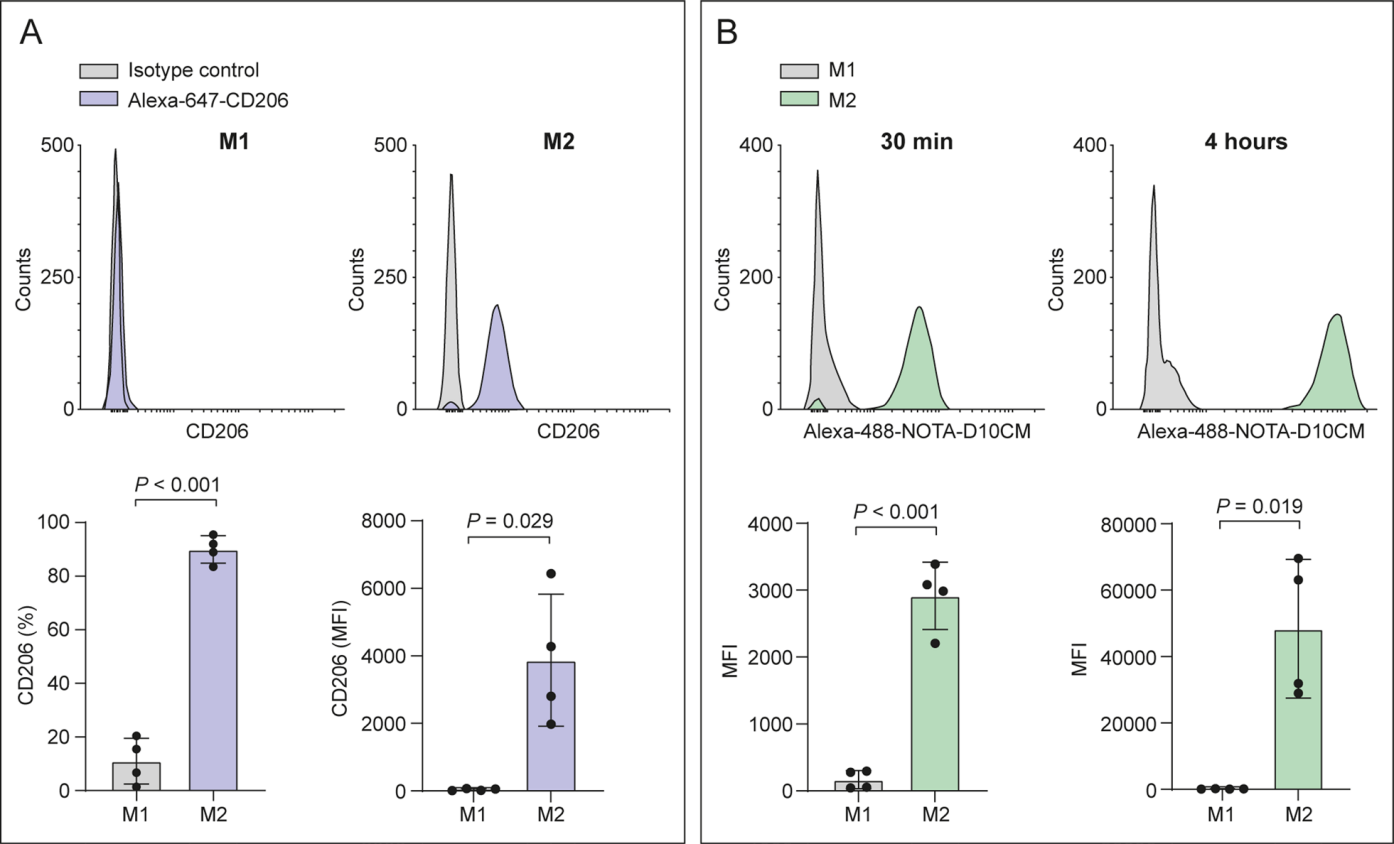
Binding of Alexa-488-NOTA-D10CM to human M1 versus M2 macrophages. **A** Peripheral blood mononuclear cells were polarized into M1 and M2 macrophages in vitro. Representative flow cytometry histograms (upper panels) and quantification (lower panels) of CD206 expression. **B** Representative flow cytometry histograms (upper panels) and quantification of Alexa-488-NOTA-D10CM binding (lower panels) to M1 and M2 macrophages after a 30-min or 4-h incubation. *MFI* mean fluorescence intensity. *P* values are from Student’s *t* test

Alexa-488-NOTA-D10CM was prepared at the concentration of 1 mg/mL. Flow cytometry confirmed M1 and M2 polarization of human monocytes enriched from PBMCs, and higher expression of CD206 by M2 macrophages than by M1 macrophages. The binding assay confirmed that more Alexa-488-NOTA-D10CM bound to M2 than to M1 macrophages (mean fluorescence intensity [MFI] at 30 min 2914.76 ± 503.10 versus 168.36 ± 137.30, respectively; *P* = 0.001), and remained bound for 4 h (Fig. 2).

**Fig. 3:**
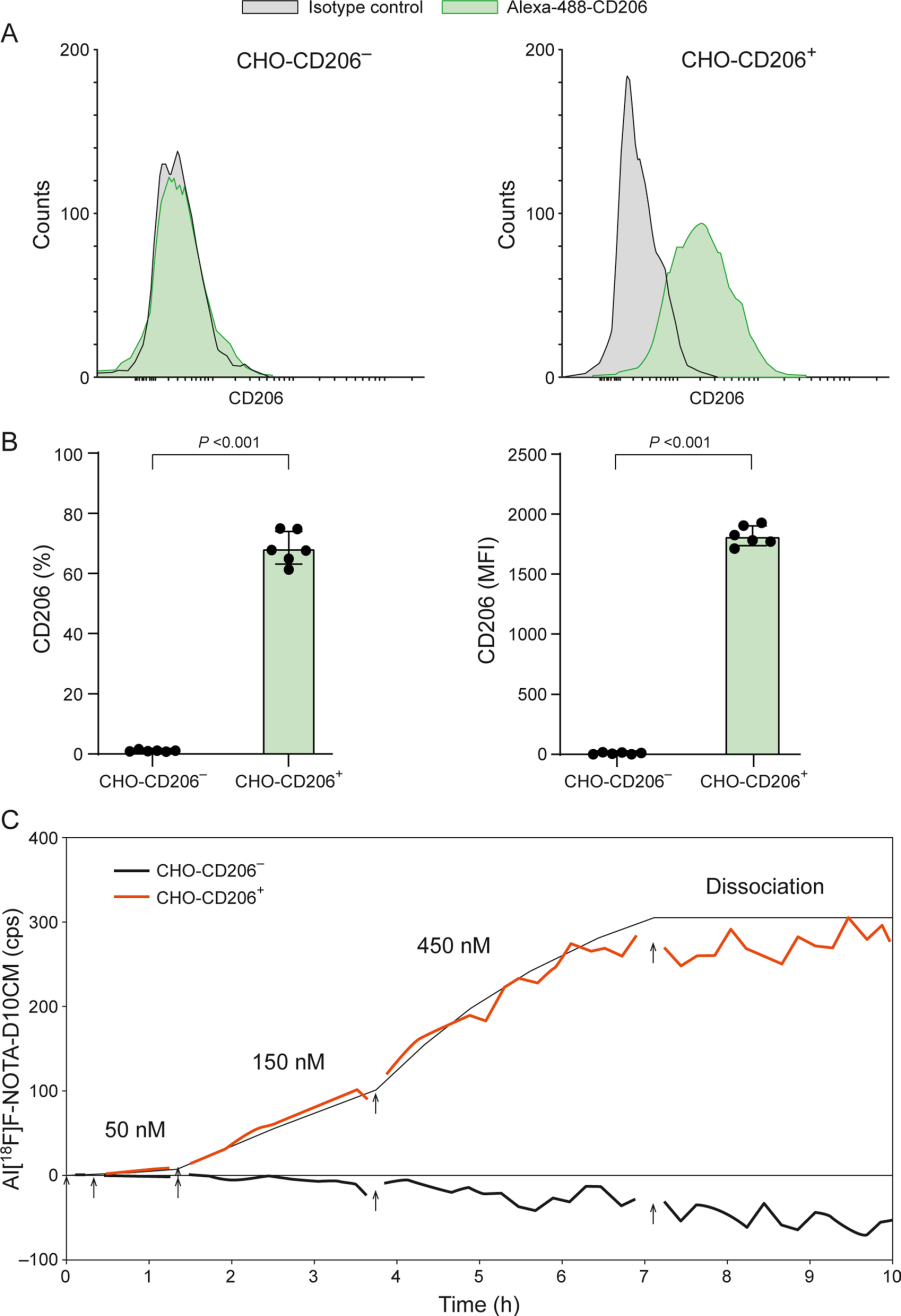
Binding of Al[^18^F]F-NOTA-D10CM to CD206^+^ versus CD206^−^ cells. **A** Representative flow cytometry histograms of mouse CD206 expression on naive Chinese hamster ovary cells (CHO-CD206^−^) and CD206-transfected cells (CHO-CD206^+^), as detected by an AlexaFluor-488 anti-mouse CD206 antibody. **B** Quantification of CD206 expression. MFI, mean fluorescence intensity. (**C**) An example of real-time binding of Al[^18^F]F-NOTA-D10CM to CHO-CD206^+^ and CHO-CD206^−^ cells, as determined using the LigandTracer instrument. *cps* counts per second. *P* values are from Student’s *t* test

Flow cytometry confirmed expression of CD206 on CHO-CD206^+^ cells, whereas CHO-CD206^−^ cells showed no expression (Fig. 3A, B). LigandTracer-based binding assays revealed that as the concentration of Al[^18^F]F-NOTA-D10CM increased from 50 to 450 nM, binding to CHO-CD206^+^ cells increased steadily, with a binding affinity of 1.83 ± 0.68 nM. No binding to CHO-CD206^−^ cells was observed (Fig. 3C).

### Uptake of Al[^18^F]F-NOTA-D10CM after MI

**Fig. 4:**
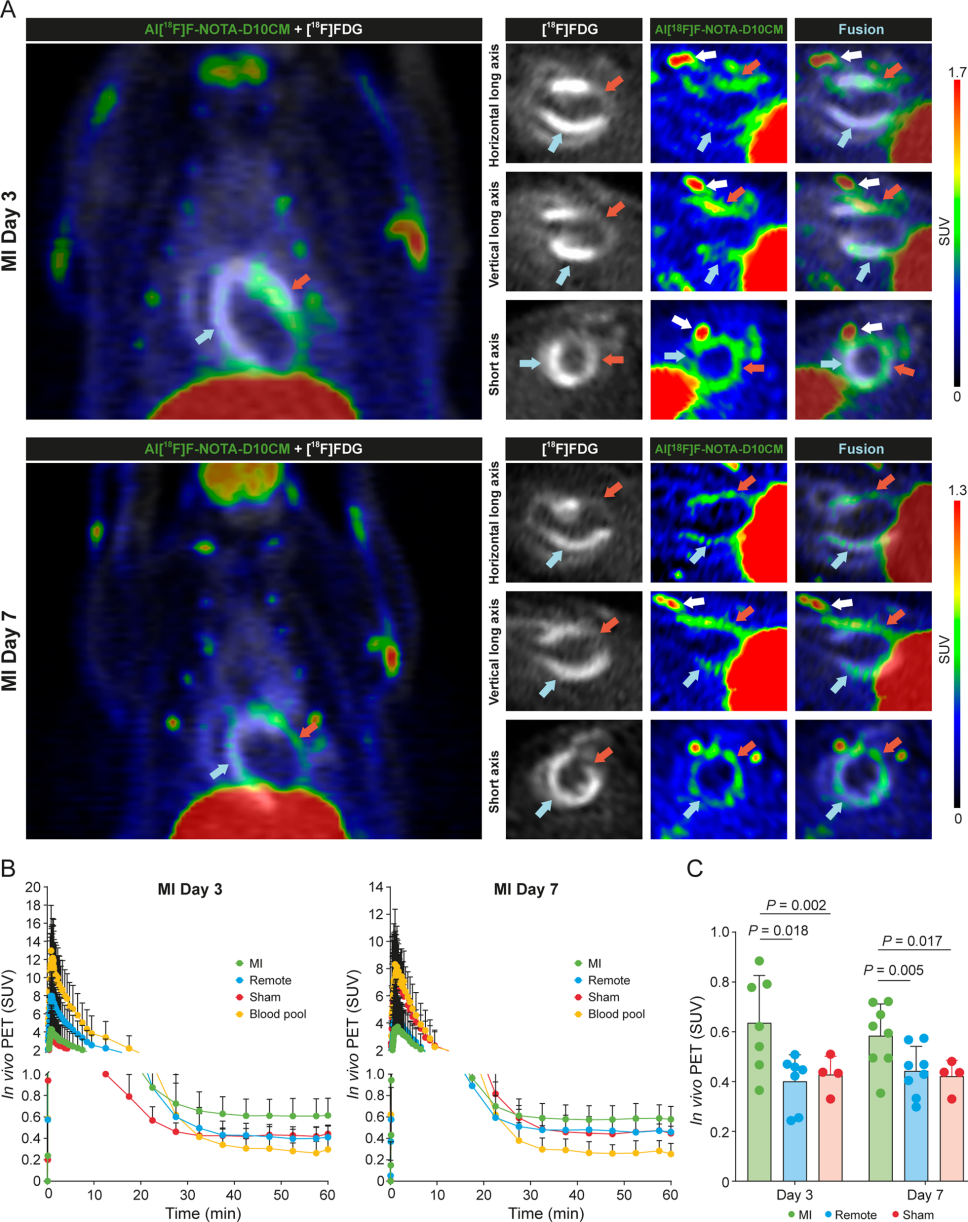
In vivo PET imaging of rats with myocardial infarction (MI). **A** Representative Al[^18^F]F-NOTA-D10CM and [^18^F]FDG PET images of rats on Day 3 and Day 7 after MI. [^18^F]FDG visualizes viable myocardium (blue arrows), while Al[^18^F]F-NOTA-D10CM shows uptake in the infarcted and border zone areas (red arrows). The white arrows denote tracer uptake in the healing surgical scar. **B** Time-activity curves of the blood pool, MI area, and remote area on Day 3 and Day 7 after MI, and of the myocardium of sham-operated rats. **C** Quantification of in vivo Al[^18^F]F-NOTA-D10CM PET/CT reveals significantly higher tracer uptake in the MI area both on Day 3 and Day 7 after MI (compared with the remote area or the myocardium of sham-operated rats). *SUV* standardized uptake value. *P* values are from one-way ANOVA

In vivo Al[^18^F]F-NOTA-D10CM PET/CT imaging was conducted to evaluate tracer uptake in the MI area (Fig. 4A, B). On Day 3, uptake of Al[^18^F]F-NOTA-D10CM in the MI area was significantly higher than in the remote myocardium (SUV 0.64 ± 0.19 vs. 0.40 ± 0.11, respectively; *P* = 0.018) or in the myocardium of sham-operated rats (SUV 0.43 ± 0.07; *P* = 0.002; Fig. 4C). Similarly on Day 7, uptake was higher in the MI area than in the remote myocardium (SUV 0.61 ± 0.16 vs. 0.45 ± 0.12, respectively; *P* = 0.047) or the myocardium of sham-operated rats (SUV 0.42 ± 0.06; *P* = 0.017). There was no difference in the Al[^18^F]F-NOTA-D10CM PET signal in the infarcted region between Day 3 and Day 7 (*P* = 0.752).

The average MI size was 31.87% ± 18.20 of the left ventricle with no significant difference between Day 3 and Day 7 (*P* = 0.282). There was no correlation between the uptake of Al[^18^F]F-NOTA-D10CM and MI size on day 3 (*r* = 0.675, *P* = 0.096) or day 7 (*r* = 0.668, *P* = 0.070).

Fitted TACs and Patlak plots are shown in Supplementary Figs. 2 and 3. The net influx rate *K*_*i*_, the transport rate constant K_1_, and the fractional blood volume V_b_ of Al[^18^F]F-NOTA-D10CM extracted from dynamic PET data are shown in Table 1. The average *K*_*i*_ and K_1_ was significantly higher in the MI area than in the remote region (*P* < 0.005), but the difference compared with sham-operated rats was not statistically significant; however, the average V_b_ in the MI area was significantly lower than in the remote region (*P* = 0.002), or in the myocardium of sham-operated rats (*P* = 0.009).

**Table 1:** Modeling parameters for the dynamic Al[^18^F]F-NOTA-D10CM PET data

Study group, region of interest	K_i_	K_1_	V_b_
Pooled MI (*n* = 15)	0.007 ± 0.002	0.012 ± 0.005	37.735 ± 15.688
Pooled remote (*n* = 15)	0.004 ± 0.002	0.010 ± 0.004	58.174 ± 13.008
Pooled sham, left ventricular wall (*n* = 8)	0.005 ± 0.002	0.012 ± 0.006	58.105 ± 17.201
*P* values			
MI vs. remote	< 0.0001	0.002	0.002
MI vs. sham	0.101	0.975	0.009

Results are expressed as mean ± SD. K_i_ = net influx rate (min^−1^); K_1_ = plasma to tissue transport rate (mL min^−1^ mL^−1^); V_b_ = fractional blood volume (mL). *P* values are from Wilcoxon test

**Fig. 5:**
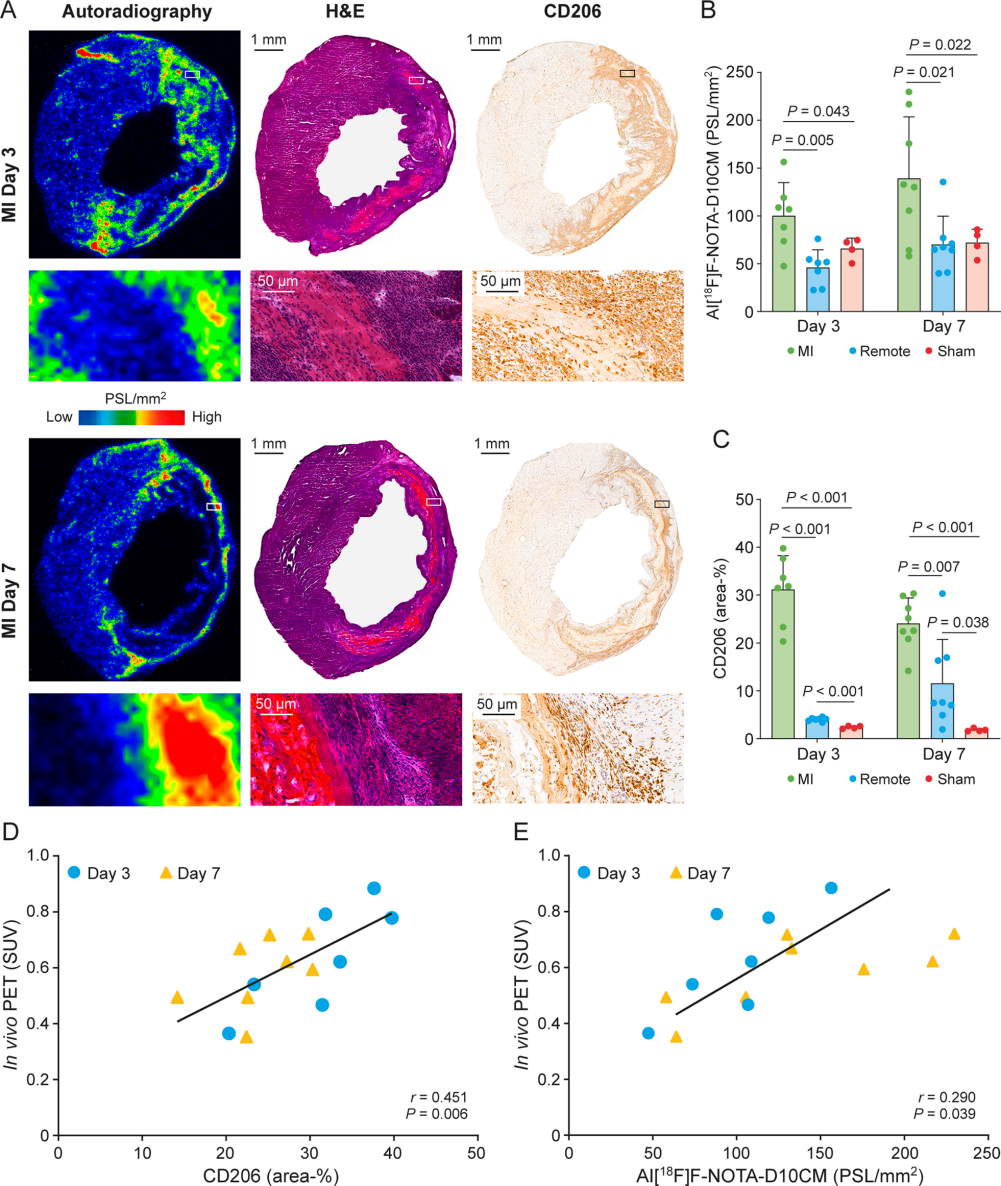
Autoradiography of myocardial Al[^18^F]F-NOTA-D10CM uptake and quantification of CD206-positive cells. **A** Representative Al[^18^F]F-NOTA-D10CM autoradiographs, hematoxylin–eosin (H&E) staining, and anti-CD206 immunohistochemical staining of the rat left ventricle on Day 3 and Day 7 after myocardial infarction (MI). Quantification of **B** Al[^18^F]F-NOTA-D10CM autoradiography and **C** CD206-positive staining reveals significantly higher signals in the infarcted and border zone areas on both Day 3 and Day 7 after MI. **D** Correlation between the Al[^18^F]F-NOTA-D10CM in vivo PET results for the infarct area and **A** the % CD206-positive stained area or **E** ex vivo autoradiography of Al[^18^F]F-NOTA-D10CM on Day 3 and Day 7 after myocardial infarction. *PSL/mm*^*2*^ photostimulated luminescence per square millimeter. *P* values are from one-way ANOVA and correlation coefficients from Pearson’s analysis

Ex vivo Al[^18^F]F-NOTA-D10CM autoradiography of the left ventricle supported the in vivo PET/CT results. Increased uptake of Al[^18^F]F-NOTA-D10CM co-localized with the MI area and immunohistochemical staining for CD206 (Fig. 5A). On Day 3, uptake in the MI area was significantly higher than in the remote area (PSL/mm^2^ 100.04 ± 34.81 vs. 45.83 ± 18.79, respectively; *P* = 0.005) or in the myocardium of sham-operated rats (65.56 ± 11.03, *P* = 0.043). Likewise, uptake on Day 7 was higher in the MI area than in the remote area (PSL/mm^2^ 139.07 ± 64.46 vs. 70.02 ± 29.70, respectively; *P* = 0.021) or in the myocardium of sham-operated rats (71.90 ± 14.16, *P* = 0.022) (Fig. 5B). There was no difference in Al[^18^F]F-NOTA-D10CM uptake in the infarcted region between Days 3 and 7 (*P* = 0.166).

The ex vivo biodistribution results are shown in Supplementary Table 1. The highest uptake of Al[^18^F]F-NOTA-D10CM at 70 min post-injection was observed in the liver, spleen, and bone marrow, with no significant differences between the MI and Sham groups. The high accumulation of Al[^18^F]F-NOTA-D10CM in liver, spleen and bone marrow were similar to our previous study in healthy rats where the CD206 immunohistochemical staining validated the co-localization [9]. Gamma counting of the whole heart showed no significant differences between the MI and Sham groups on either on Day 3 or 7.

### Detection of CD206 in rat MI areas

**Fig. 6:**
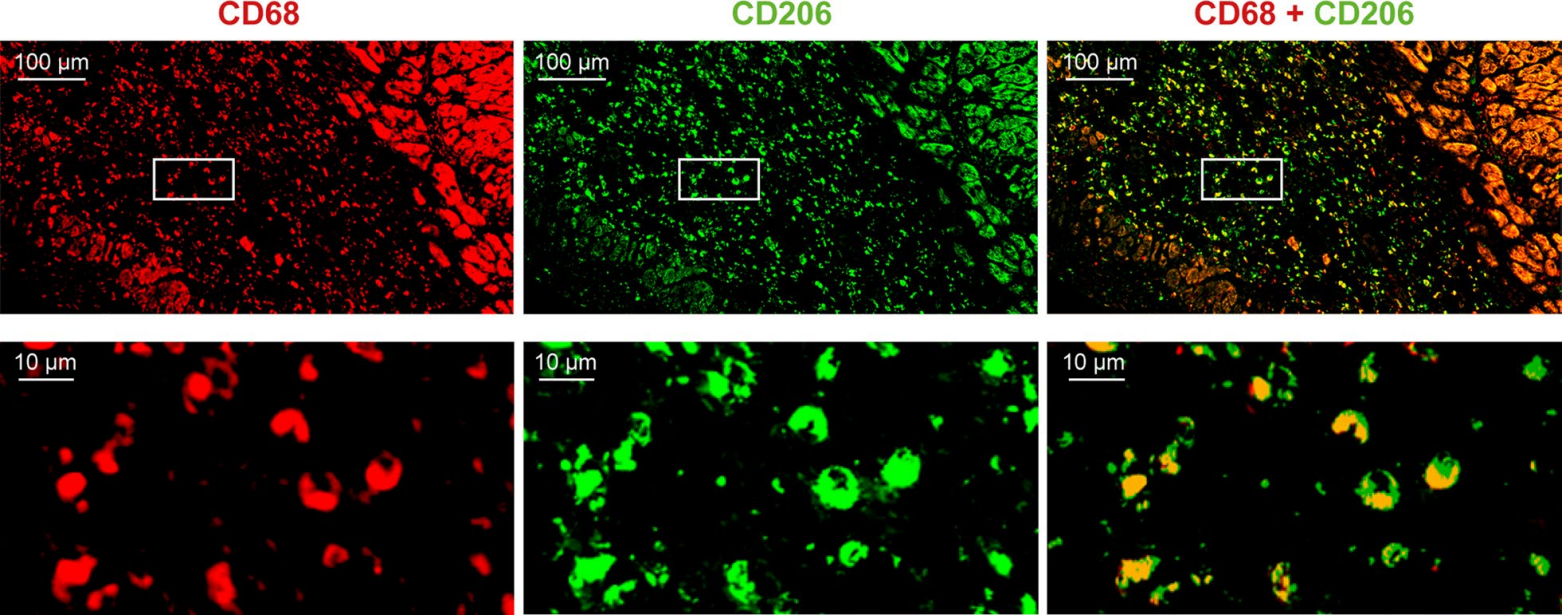
CD206 and CD68 in rat myocardial tissue after infarction. Double immunofluorescence staining of a rat left ventricle reveals co-localization of CD68-positive staining and macrophage mannose receptor CD206-positive staining in the injured area on Day 7 after MI

All rats studied after LAD-ligation showed histological signs of MI, as well as CD206-positive macrophages in the infarcted area (Fig. 5A). The CD206-positive area-% in the MI area was significantly higher than that in the remote area or in the myocardium of sham-operated rats both on Day 3 (31.14 ± 7.08 vs. 4.02 ± 0.38 vs. 2.37 ± 0.29, respectively; *P* < 0.001) and Day 7 (24.07 ± 5.29 vs. 10.77 ± 9.90, *P* = 0.007, vs. 1.80 ± 0.31, respectively; *P* < 0.001) (Fig. 5C). The CD206-positive area-% in the MI region tended to be higher on Day 3 than on Day 7 (*P* = 0.053). Double immunofluorescence staining with Alexa-594-CD68 and Alexa-488-CD206 antibodies confirmed co-localization of CD206 staining and CD68-positive macrophages in the MI area (Fig. 6).

There was a positive correlation between the CD206-positive area-% and in vivo PET SUV (*r* = 0.451, *P* = 0.006; Fig. 5D) and ex vivo autoradiography PSL/mm^2^ (*r* = 0.290, *P* = 0.039) values in the infarcted region on Days 3 and 7 post-MI (Fig. 5E, Supplementary Fig. 4).

### Specificity of Al[^18^F]F-NOTA-D10CM for the MI area

**Fig. 7:**
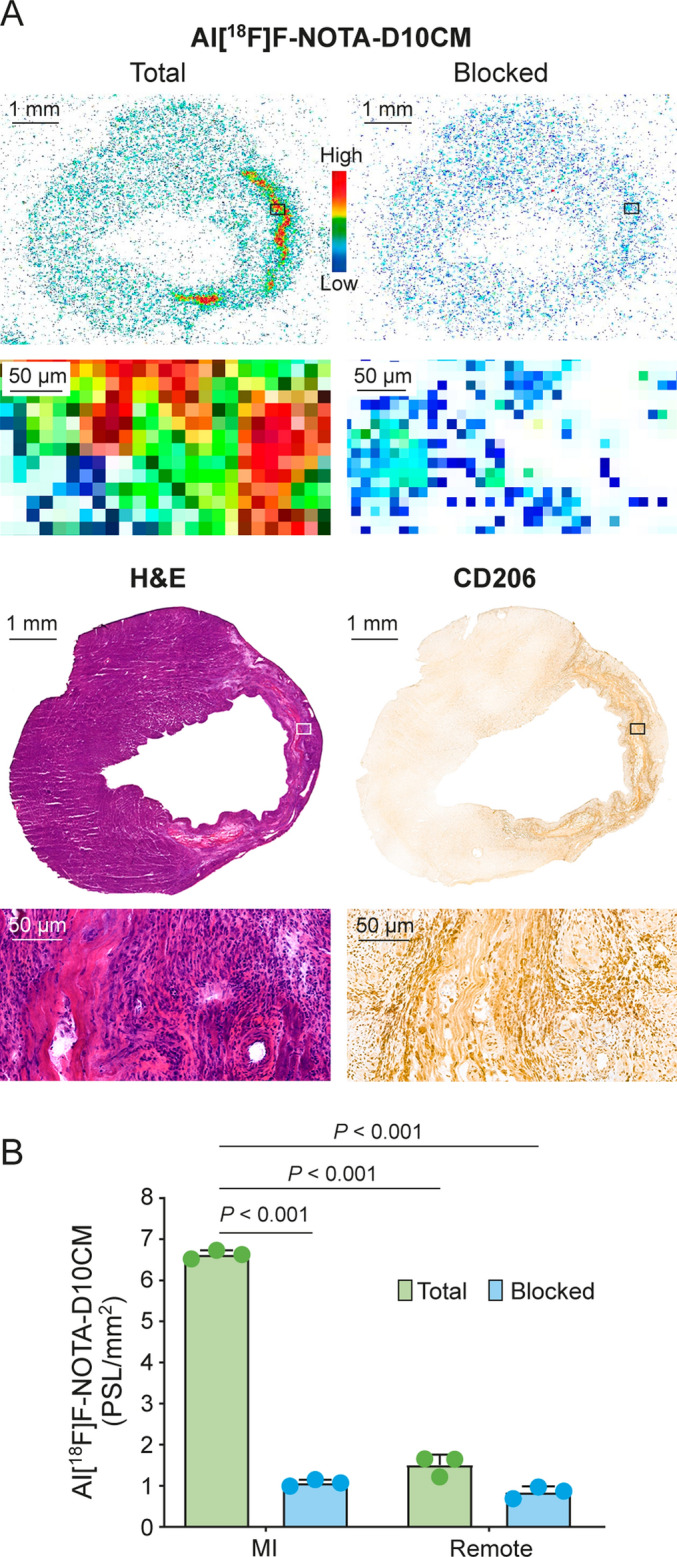
In vitro competitive study confirming Al[^18^F]F-NOTA-D10CM binding specificity. **A** Representative Al[^18^F]F-NOTA-D10CM autoradiographs, and hematoxylin–eosin (H&E) and anti-CD206 staining of adjacent cryosections of the left ventricle post-myocardial infarction (MI). **B** Quantification of Al[^18^F]F-NOTA-D10CM binding without and with co-incubation with an excess of unlabeled NOTA-D10CM. *PSL/mm*^*2*^ photostimulated luminescence per square millimeter. *P* values are from Student’s *t* test

The in vitro competitive study of adjacent left ventricle sections revealed that co-incubation with an excess of unlabeled NOTA-D10CM reduced binding of Al[^18^F]F-NOTA-D10CM in the MI area by 85.21% ± 2.51 (*n* = 3) (Fig. 7).

## Discussion

In this study, we demonstrate that macrophage mannose receptor CD206-targeted PET imaging using the ^18^F-labeled mannosylated dextran derivative Al[^18^F]F-NOTA-D10CM can detect CD206-positive macrophages at an early time point after experimental MI. Tracer uptake correlates with the amount of CD206-positive macrophages in the infarcted myocardium. These results provide evidence that Al[^18^F]F-NOTA-D10CM PET is a potential tool to study immune response after MI.

The U.S. Food and Drug Administration has approved macrophage mannose receptor CD206-targeted mannosylated dextran derivative ^99m^Tc-Tilmanocept single-photon emission CT imaging for lymph node mapping [8]. Previously, we developed a CD206-targeted PET tracer, Al[^18^F]F-NOTA-D10CM (an ^18^F-radiolabeled NOTA-conjugated mannosylated dextran derivative) that can be produced with a high radiochemical yield, and that shows rapid blood clearance, excellent in vivo stability, and specific accumulation in CD206-rich tissues [12]. Our subsequent validation study in mice demonstrated that Al[^18^F]F-NOTA-D10CM PET/CT can identify inflammatory foci after intravenous administration, and can map lymph node activation after intradermal injection [13].

Several CD206-targeted PET imaging agents have been developed and investigated in the context of various inflammatory diseases and cancer, in addition to detection of lymph node activation [6, 7, 19–23]. These imaging agents include mannosylated vesicles (^64^Cu-labeled MAN-LIPs) [19], antibody or antibody fragment-based agents (^18^F-FB-anti-MMR sdAb) [20], [^68^Ga]Ga-NOTA-anti-MMR-sdAb [21], and ^68^Ga-NOTA-anti-MMR Nb [6, 7]), peptide-based agents ([^68^Ga]RP832c) [22], mannosylated human serum albumin (^68^Ga-NOTA-MSA [4], ^68^Ga-MSA [7]), a mannose derivative ([^18^F]FDM) [6], and [^68^Ga]Ga-tilmanocept [23]). [^68^Ga]Ga-NOTA-anti-MMR-sdAb has undergone a Phase I clinical study to assess the radiation dose, and the ability to detect protumorigenic macrophages in solid tumors; the trial showed promising results and is continuing as Phase II clinical trial [5].

Previously, CD206-targeted PET imaging study using ^68^Ga-labeled 1,4,7-triazacyclononane-1,4,7-triacetic acid-conjugated nanobody (^68^Ga-NOTA-anti-MMR Nb) was conducted in mouse and rat models with permanent LAD ligation [9]. The imaging agent comprised an anti-MMR nanobody (~ 15 kDa) derived from a camelid heavy chain-only antibody specific for human and mouse CD206, which was radiolabeled with short-lived ^68^Ga (t_1/2_ = 68 min). Higher focal radioactivity signals were detected in MI rats and wild-type mice MI than in the myocardium of sham-operated animals and CD206-knockout mice. The PET-image derived infarct-to-remote ratio in rats 7 days after LAD ligation was 1.3 ± 0.2. When using Al[^18^F]F-NOTA-D10CM PET, the average infarct-to-remote ratio was 1.5 ± 0.3 (pooled data from Day 3 and Day 7 post-MI). Despite having different structures, both our Al[^18^F]F-NOTA-D10CM and the previously described ^68^Ga-NOTA-anti-MMR Nb specifically detect CD206; however, they target different binding domains. The mannosylated dextran sugar of Al[^18^F]F-NOTA-D10CM attaches to carbohydrate recognition domain 4 (CRD-4) [24], whereas the ^68^Ga-NOTA-anti-MMR Nb binds to a peptide or protein recognition domain, i.e., the collagen-binding fibronectin type II domain [25]. Both Al[^18^F]F-NOTA-D10CM and ^68^Ga-NOTA-anti-MMR Nb accumulate in the liver and spleen, i.e., organs known to express CD206; however, the ^68^Ga-NOTA-anti-MMR Nb also shows high retention in the kidneys, which is typical for peptides and small proteins such as antibody fragments [26]. The binding affinities of Al[^18^F]F-NOTA-D10CM (1.83 nM in mouse CD206-positive CHO cells) and ^68^Ga-NOTA-anti-MMR Nb (1.8 nM to human CD206) to CD206 are comparable [20].

Significantly, we confirmed higher uptake of Al[^18^F]F-NOTA-D10CM in the MI area by autoradiography, which correlated with CD206-positive macrophages. Although a distinct difference in Al[^18^F]F-NOTA-D10CM uptake between day 3 and day 7 post-MI was not observed, this outcome likely reflects underlying biological variability in the healing process rather than a limitation of the tracer itself. The ability of Al[^18^F]F-NOTA-D10CM to detect CD206-positive macrophages as early as day 3 post-MI underscores its potential for early, non-invasive assessment of inflammation after MI*.* In addition, the uptake of Al[^18^F]F-NOTA-D10CM in the MI area was independent of infarct size. Furthermore, the competitive binding assay with a molar excess of unlabeled NOTA-D10CM reduced uptake of Al[^18^F]F-NOTA-D10CM in the MI area by ~ 85%, confirming that the tracer uptake is specific. Although CD206-targeted imaging of MI has been attempted before, a tracer based on a radionuclide with a longer physical half-life, ^18^F (t_1/2_ = 109.7 min), and lower positron energy than ^68^Ga provides better spatial resolution for PET imaging, which is important for visualization of small structures [27].

Compared to previously reported CD206-targeted tracers, Al[^18^F]F-NOTA-D10CM offers several notable advantages. This tracer selectively binds to CD206 expressed on M2 macrophages and demonstrates favorable pharmacokinetics, characterized by rapid clearance from the blood circulation and selective tissue accumulation. These distribution patterns are consistent with CD206-mediated binding, and are corroborated by anti-CD206 immunohistochemical staining conducted in this study. Importantly, because CD206 expression reflects a functional macrophage phenotype rather than a specific immune cell lineage—unlike tracers that selectively target T cells or activated neutrophils—Al[^18^F]F-NOTA-D10CM may offer broader applicability across diverse stages of immune response. In comparison to peptide-based CD206-targeted tracers, mannosylated dextran platform employed here confers several advantages including enhanced in vivo stability, reduced susceptibility to enzymatic degradation, and increased binding avidity. The latter arises from multivalent mannose presentation, achieved through the attachment of 17 mannose moieties to a single D10CM molecule [12]. Moreover, while peptide-based tracers frequently exhibit elevated renal uptake due to their small molecular size, dextran-based constructs such as Al[^18^F]F-NOTA-D10CM display more favorable biodistribution profiles for imaging inflammatory processes. Finally, the dextran scaffold contributes to the overall biocompatibility and safety of the tracer, supporting its suitability for clinical translation. From a radiopharmaceutical production standpoint, Al[^18^F]F-radiolabeling chemistry offers a straightforward approach and that is highly compatible with fully automated manufacturing process [28], thereby supporting its clinical application and potential for widespread implementation.

Some studies suggest that targeting the macrophage mannose receptor CD206 can be used as a tool for monitoring inflammatory status. Examples include predicting the severity of inflammation, or the prognosis for inflammatory diseases [4, 29, 30]. Considering its anti-inflammatory and reparative properties, macrophage mannose receptor CD206 might be a marker for monitoring the inflammation resolution process during healing post-MI. Involvement of CD206-positive macrophages after MI is thought to promote cardiac repair by regulating the activation of fibroblasts [11]. Siraishi and coworkers demonstrated this by ablating tribbles pseudokinase 1 (TRIB1) in mice, which depleted M2 macrophages after MI, thereby causing impaired fibroblast activation in the infarct, leading to an increased rate of myocardial rupture [11]. The time-course of Al[^18^F]F-NOTA-D10CM uptake intensity was consistent with the M2 macrophage response, which peaked at Day 7 post-MI, as shown by Varasteh and colleagues [9]. In our study, however, the tracer visualized the onset of M2 macrophage infiltration as early as day 3 post-MI. Interestingly, Mouton and co-workers found a similar trend where proliferation of M2 macrophages started on Day 3 post-MI [31].

We recognize that our study has some limitations. Prominent expression of CD206 in the liver, leading to high accumulation of Al[^18^F]F-NOTA-D10CM, may have a spillover effect that could affect quantification of the myocardium in PET images, especially the inferior wall of the left ventricle. Careful placement of ROIs in the apex and inferior wall can minimize this. Furthermore, autoradiography confirmed the in vivo results, which can be combined directly with histology and CD206 staining. Additional time points post-MI, such as before Day 3 and after Day 7, could provide additional information about the dynamics of the response, as reflected by variations in expression of anti-inflammatory macrophages. Furthermore, our results may not be generalizable in setting of ischemia–reperfusion injury. Another limitation is that we failed to perform an in vivo PET blocking study; this is because the blood-rich heart also accumulates an excess of free Al[^18^F]F-NOTA-D10CM, which circulates in the blood after the blockage; however, the in vitro competitive displacement assay using tissue cryosections demonstrated the binding specificity of Al[^18^F]F-NOTA-D10CM for the MI area.

## Conclusion

Al[^18^F]F-NOTA-D10CM PET detects overexpression of CD206 after ischemic myocardial injury, making it a suitable biomarker for detecting M2-type macrophages associated with inflammatory process post-MI.

## Supplementary Information

Additional file 1.

## Data Availability

The original data of the work can be obtained from Prof. Anne Roivainen upon rational request.

## Abbreviations

%ID/g: Percentage of injected radioactivity dose per gram of tissue
[^18^F]FDG: 2-Deoxy-2-[^18^F]-fluoro-d-glucose
Al[^18^F]F-NOTA-D10CM: Aluminum fluoride-18-labeled 1,4,7-triazacyclononane-1,4,7-triacetic acid-conjugated mannosylated dextran derivative
ANOVA: Analysis of variance
CD206: Cluster of differentiation 206
CD68: Cluster differentiation 68
CHO: Chinese hamster ovary
cps: Counts per second
CT: Computed tomography
D10CM: 21.3 KDa mannosylated dextran with 7 cysteines and 19 mannoses
H&E: Hematoxylin–eosin
HEPES: *N*-(2-Hydroxyethyl)-piperazine-*N’*-(2-ethanesulfonic acid)
HPLC: High-performance liquid chromatography
i.p.: Intraperitoneal
i.v.: Intravenous
K_1_: Transport rate
K_D_: Dissociation constant
K_i_: Net influx rate
LAD: Left anterior descending
MFI: Mean fluorescence intensity
MI: Myocardial infarction
NOTA: 1,4,7-Triazacyclononane-1,4,7-triacetic acid
PBMC: Peripheral blood mononuclear cell
PBS: Phosphate-buffered saline
PET: Positron emission tomography
PSL/mm^2^: Photostimulated luminescence per square millimeter
ROI: Region of interest
s.c.: Subcutaneous
SD: Standard deviation
SUV: Standardized uptake value
TAC: Time-activity curve
TFA: Trifluoroacetic acid
V_b_: Fractional blood volume
