# Supplementary material for: Macrophage mannose receptor CD206-targeted PET imaging in experimental acute myocardial infarction
Source: EJNMMI Res. 2025 Jun 4;15:66. doi: 10.1186/s13550-025-01254-2 (PMC12137864; doi:10.1186/s13550-025-01254-2)
Supplement: Supplementary file 1 — Additional file 1. [file 13550_2025_1254_MOESM1_ESM.pdf]

Supplementary Material

Macrophage mannose receptor CD206-targeted PET imaging in experimental acute myocardial infarction

Putri Andriana[1], Senthil Palani[1], Heidi Liljenbäck[1,2], Imran Iqbal[1], Vesa Oikonen[1], Jenni Virta[1], Konstantina Makrypidi[3], Johan Rajander[4], Erika Atencio Herre[1], Aino Suni[1], Sirpa Jalkanen[5,6], Juhani Knuuti[1,6,7], Luisa Martinez-Pomares[8], Ioannis Pirmettis[3], Xiang-Guo Li[1,6,7,9], Antti Saraste[1,7,10] and Anne Roivainen[1,2,6,7*]

[1]Turku PET Centre, University of Turku, Turku, Finland

[2]Turku Center of Disease Modeling, University of Turku, Turku, Finland

[3]Institute of Nuclear and Radiological Science and Technology, Energy and Safety, NCSR "Demokritos", Athens, Greece

[4]Turku PET Centre, Accelerator Laboratory, Åbo Akademi University, Turku, Finland

[5]MediCity Research Laboratory, University of Turku, Turku, Finland

[6]InFLAMES Research Flagship, University of Turku, Turku, Finland

[7]Turku PET Centre, Turku University Hospital, Turku, Finland

[8]School of Life Sciences, University of Nottingham, Nottingham, UK

[9]Department of Chemistry, University of Turku, Turku, Finland

[10]Heart Center, Turku University Hospital and University of Turku, Turku, Finland

*Correspondence: Professor Anne Roivainen, PhD, Turku PET Centre, Kiinamyllynkatu 4-8, FI-20520 Turku, Finland; Phone: +35823132862; E-mail: anne.roivainen@utu.fi

Supplementary Table 1 *Ex vivo* biodistribution of Al[^{18}F]F-NOTA-D10CM at 70 min post-intravenous injection into rats

Tissue	MI		Sham	
	Day 3 (*n* = 7)	Day 7 (*n* = 7)	Day 3 (*n* = 4)	Day 7 (*n* = 4)
Blood	0.004 ± 0.001	0.006 ± 0.008	0.003 ± 0.001	0.002 ± 0.001
Bone (skull)	0.158 ± 0.074	0.179 ± 0.036	0.242 ± 0.038	0.218 ± 0.067
Bone marrow (femur)	2.624 ± 0.495	2.923 ± 0.907	2.549 ± 0.902	2.998 ± 1.258
Brain (intact)	0.008 ± 0.000	0.002 ± 0.001	0.001 ± 0.000	0.001 ± 0.001
Heart	0.231 ± 0.089	0.319 ± 0.154	0.340 ± 0.052	0.242 ± 0.040
Intestine (large)	0.266 ± 0.052	0.281 ± 0.111	0.335 ± 0.072	0.365 ± 0.227
Intestine (small)	0.369 ± 0.077	0.381 ± 0.203	0.469 ± 0.069	0.273 ± 0.184
Kidneys	0.478 ± 0.124	0.484 ± 0.155	0.545 ± 0.134	0.496 ± 0.149
Liver	5.734 ± 1.463	5.118 ± 1.392	6.690 ± 3.015	6.853 ± 4.707
Lungs	0.217 ± 0.053	0.154 ± 0.081	0.205 ± 0.039	0.133 ± 0.040
Muscle	0.030 ± 0.011	0.024 ± 0.012	0.038 ± 0.005	0.023 ± 0.004
Pancreas	0.449 ± 0.140	0.531 ± 0.168	0.661 ± 0.163	0.491 ± 0.196
Spleen	3.072 ± 0.750	2.508 ± 0.461	2.923 ± 1.083	2.603 ± 1.270
Testis	0.054 ± 0.016	0.051 ± 0.021	0.086 ± 0.007	0.058 ± 0.022
Thymus	0.107 ± 0.044	0.079 ± 0.062	0.180 ± 0.014	0.064 ± 0.017

Results are expressed as the percentage of the injected radioactivity dose per gram of tissue (mean ± SD).

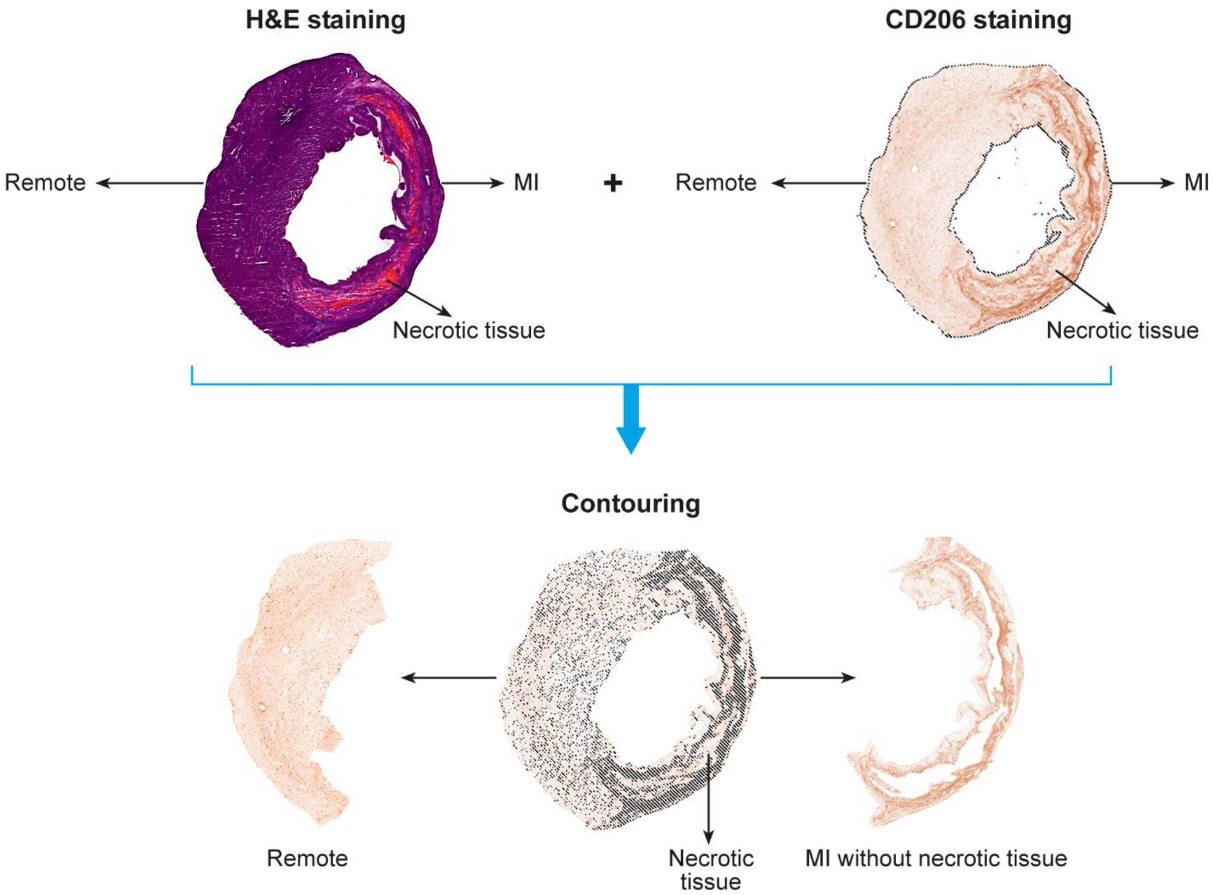

Supplementary Fig, 1 Analysis of CD206 immunohistochemical staining of the rat left ventricle cryosections after myocardial infarction (MI). The MI area, the remote area, and necrotic tissue were visually observed in histological hematoxylin-eosin (H&E) staining, their contours were delineated, and copied onto the anti-CD206 stained adjacent section. The analysis was performed using GIMP (version 2.10.24) and ImageJ (version 1.52n) software.

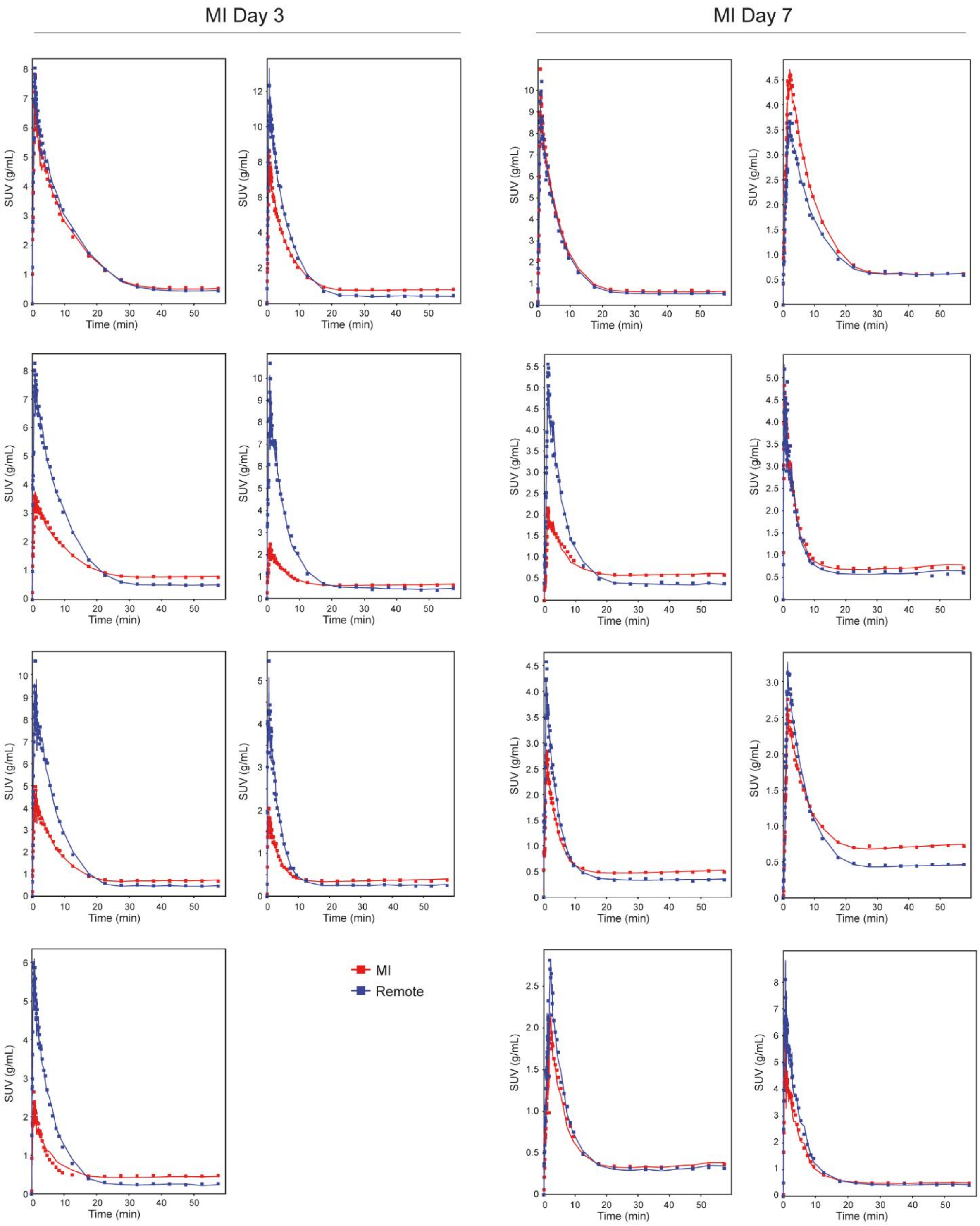

Supplementary Fig. 2 Kinetics of intravenously injected Al[^{18}F]F-NOTA-D10CM in rats with myocardial infarction (MI). Fitted time-activity curves of the MI and remote areas in each rat on Day 3 and Day 7 after permanent coronary artery ligation.

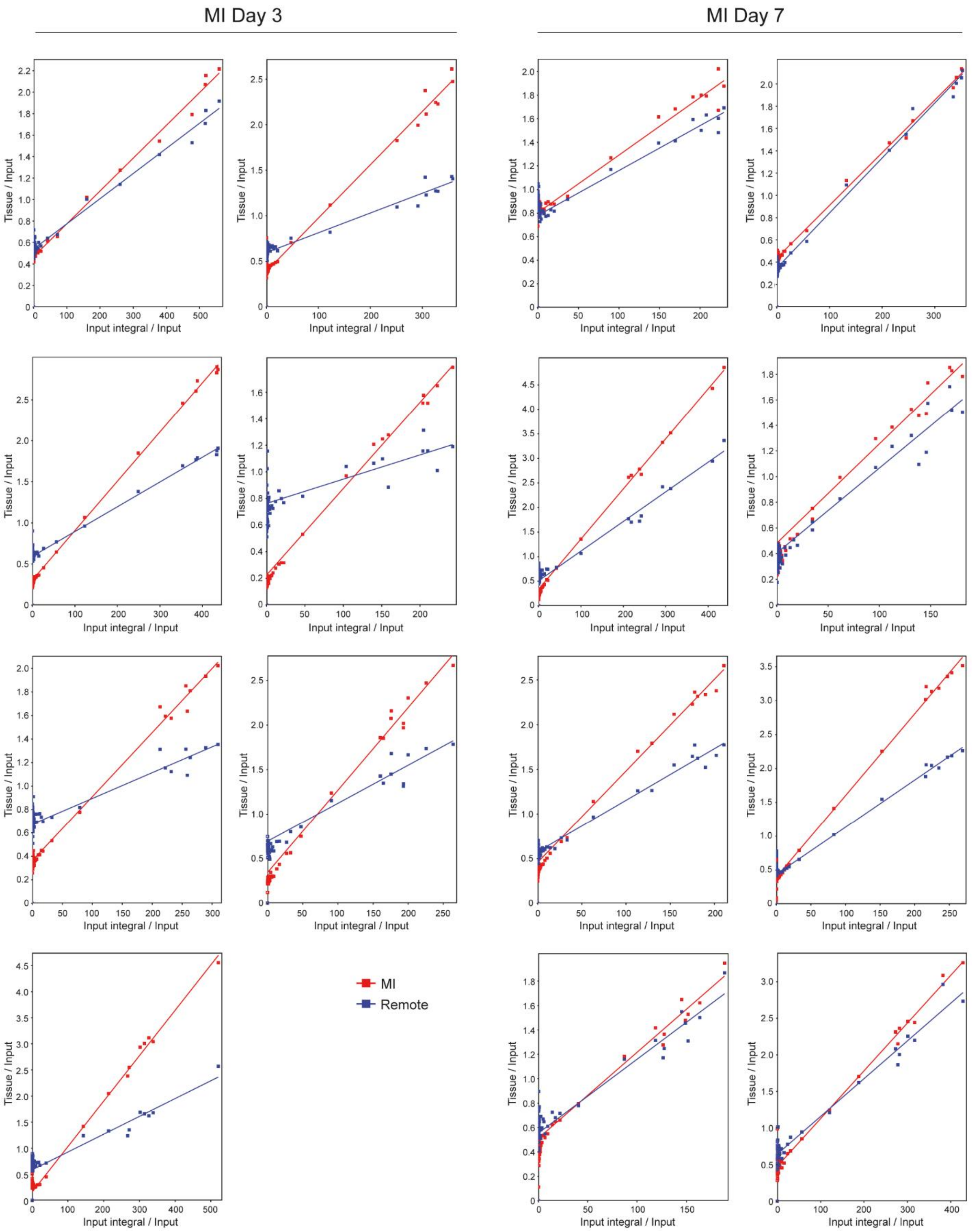

Supplementary Fig. 3 Graphical analysis of Al[^{18}F]F-NOTA-D10CM uptake in rats with myocardial infarction (MI). Patlak plots of the MI and remote areas in each rat on Day 3 and Day 7 after permanent coronary artery ligation.

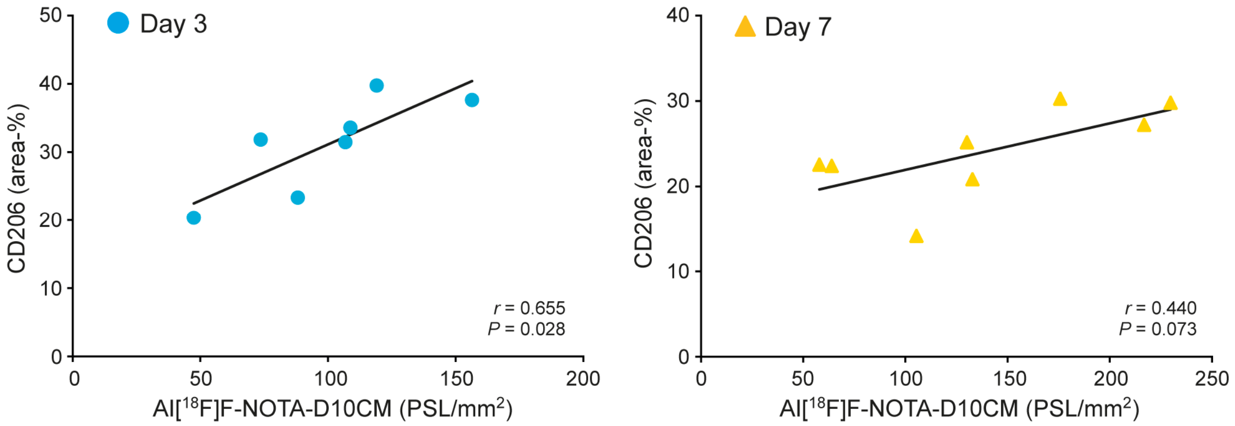

Supplementary Fig. 4 Association of Al[^{18}F]F-NOTA-D10CM and CD206. Correlation between CD206-positive staining (area-%) and Al[^{18}F]F-NOTA-D10CM uptake (autoradiography) by the infarction region on Day 3 and Day 7 after myocardial infarction. PSL/mm^2 = photostimulated luminescence per square millimeter. Correlation coefficients are from Pearson's analysis.
